# From recreational to income-generating opportunities: assessment of public preferences for non-wood forest products in the Czech Republic

**DOI:** 10.3389/fnut.2023.1193203

**Published:** 2023-09-18

**Authors:** Ratna Chrismiari Purwestri, Miroslava Hochmalová, Miroslav Hájek, Petra Palátová, Vilém Jarský, Diana Carolina Huertas-Bernal, Mayang Christy Perdana, Sandra Paola García-Jácome, Betha Lusiana, Marcel Riedl

**Affiliations:** ^1^Faculty of Forestry and Wood Sciences, Czech University of Life Sciences Prague, Praha–Suchdol, Czechia; ^2^Faculty of Environmental Sciences, Czech University of Life Sciences Prague, Praha-Suchdol, Czechia; ^3^CIFOR-ICRAF, Bogor, West Java, Indonesia

**Keywords:** non-wood forest product, preference, policy, recreation, income-generating

## Abstract

With the alarming increase in dying trees and massive logging in the Czech forests due to bark beetle infestation, the collection of non-wood forest products, a beneficial recreational activity in the Czech Republic, is now being promoted as an alternative to wood provisioning services. This paper aims to present findings on the non-wood forest product preferences in the country as part of a baseline assessment for promoting the usage. This study relied on the 2019 national survey data of public preferences in collecting forest berries, mushrooms, honey, and medicinal herbs. K-means cluster analysis was employed to classify the respondents. A binary logistic regression with a conditional forward approach was employed to identify the potential predictors of the high preference for each non-wood forest product. Data from 1,050 online respondents were included, and two groups of respondents were clustered based on their preferences for the entire non-wood forest, i.e., higher and lower utilization. The regression analysis revealed that frequent forest visitors were the primary predictor of high utilization of all non-wood forest products (between 1.437 to 4.579 odd ratios), in addition to age, gender, and location of the forest property. By clustering the respondents based on the high and low preferences in utilizing non-wood forest products, the promotion of this service, from recreational to potential livelihood activities and economic benefits, can be better targeted, e.g., target customer, infrastructure development in the location with high preferences, scenarios based on the type of owners (municipal or private forest owners), which in accordance to the national forest policy and laws, and, at the same time, maintain the ecological stability.

## 1. Introduction

Forests provide various ecosystem services essential for human wellbeing. Besides wood, other forest provisioning services are food, fresh water, medical sources, and other raw materials (1, 2). Since time immemorial, the collection of non-wood forest products has been combined with activities like hunting, fishing, and gathering (3–5). Moreover, according to Schulp et al. (6), across the whole of Europe is a wide variety of wild edible food products collected by people: game (38 species), mushrooms (27 species), and vascular plants (81 species). Previously, the demand for wild foods was high, especially in the age of famine and scarcity (7, 8). However, game hunting and fishing or mushroom/berry collection recently became popular leisure activities in Central European countries (9). Unfortunately, in recent years the consumption of edible wild food tended to decrease as affected by different factors. The most significant cause is certain kinds of plants no longer grow in their places of origin (10); the second factor is increasing urbanization (11), the loss of human knowledge of plants, losing touch with nature (12), and damage of the plant quality by anthropological influence (13).

For decades, there has been an overemphasis on timber-oriented management; thus, the importance of the non-wood forest provisioning service was overlooked. Lack of attention to the value of non-wood forest products inhibits the forest owners’ management opportunities and competitiveness, although their management has a less ecologically negative influence than timber harvesting (14, 15). Currently, the priority of the European Union (EU) forest policy is to ensure the multifunctionality of forests, i.e., to ensure the wide-spectrum provision of material and non-material services to satisfy human needs, thus providing society with a comprehensive list of environmental, social, and economic benefits (16–18). It stands to reason that if we want to achieve a multifunctional forest economy, integrating the entire spectrum of the ecosystem services into political decision-making processes is essential. Their advancement may help to achieve common EU forest policy goals.

Information from the evaluation and monitoring of ecosystem services provides essential information about natural resources. It enables assessment of their worth, allowing the decision-makers to re-examine the environmental values (14–18). Few studies have used non-economic methods to explore the ecosystem service problems caused by socio-cultural values, attitudes, and beliefs (19–22). Since the assessment of ecosystem services is determined by analyzing the impact of ecosystems and biodiversity on human wellbeing, it is necessary to understand how society benefits from nature. Moreover, knowledge of society’s motives and preferences for non-wood forest products can be very effective for forest owners in forest management planning (22), e.g., in mitigating the difficulty of focusing on suitable stand diversity choices earlier. The obtained values do not use the ecosystem service’s monetary languages, for instance, by ranking the utilization of the services, preferences, expectations, or by the performance level perception (23–25).

Many studies examined the promotion of non-wood forest product usage from the point of view of property owners and managers, which involved the company’s preferences, business capital, potential market, infrastructure, or supporting national policy (26–28). In the EU, a total of 25% of the households participated in collecting or using non-wood forest products, where usage depends on the traditions, political regulations, and economic conditions in each country (15). When comparing the regions in Europe, there are varying perspectives on the significance of forests. In the northern and western regions of Europe, there is a stronger connection between forests and economic considerations, with a greater emphasis on the multifunctional use of forests. Additionally, collecting forest fruits is more popular among residents of Central and Northern Europe compared to the southern regions (29). The southern parts of Europe collected other various types of non-timber goods, such as wild herbs, aromatic plants, medicinal plants, chestnuts or oak corks (30, 31).

**Figure 1:**
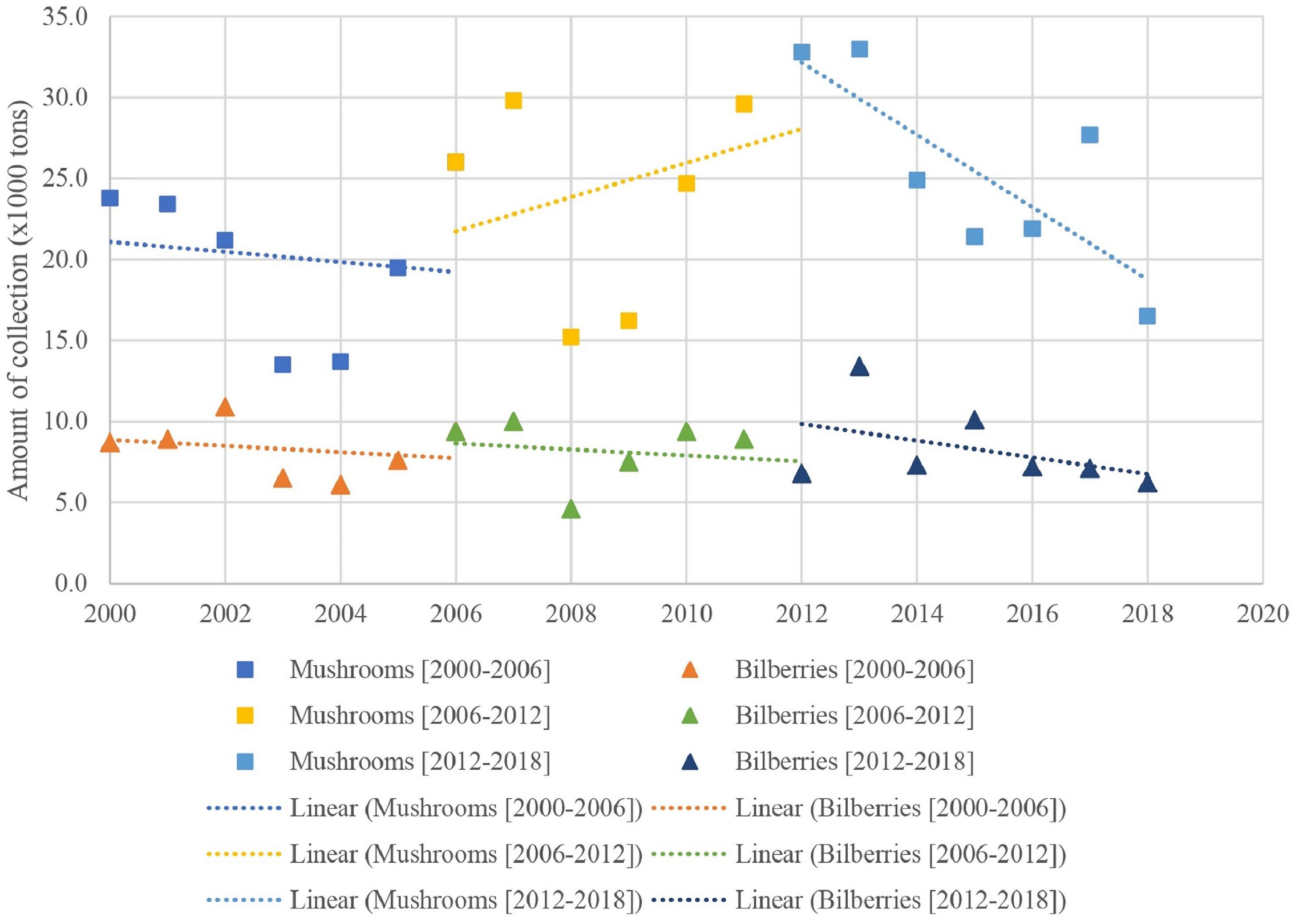
Collection of mushrooms and bilberries from 2000 to 2018. Figure is modified from Šišák et al. (35).

In the Czech Republic, the collection of mushrooms and berries is among the favorite forest recreational activities, as annually reported by the Ministry of Agriculture of the Czech Republic (32, 33). However, they are not economically valued (34, 35). Bilberries (*Vaccinium myrtillus* L.) are the most gathered berries in the country, followed by raspberries (*Rubus idaeus* L.). Other collected forest berries are blackberries (*Rubus fruticosus* L.), cowberries (*Vaccinium vitis-idaea* L.), and elderberries (*Sambucus nigra* L.). Meanwhile, mushrooms are reported as the total amount collected and are not differentiated by species. As the Czech forests have also experienced the adverse effect of climate change, i.e., drought followed by the exponential increase of bark beetles and their infestation into coniferous trees, especially in the southern and eastern parts of the country (32, 36–38), mushroom and berry collection tended to decrease. Figure 1 depicts the modified trend of mushrooms and bilberries (35) from 2000 to 2018 (32, 33).

With the alarming increase in dying trees and massive logging in the Czech forests, the promotion of other forest ecosystems is recommended as an alternative to wood provisioning services. To date, gathering non-wood forest products is still not economically valuable for Czech forest owners and managers. The research question of the paper is: what are the factors influencing society’s high preferences for non-wood forest products to promote the ecosystem service, from primarily focusing on public recreation to an income-generating activity?

The hypothesis posits that the factors leading to society’s high preference for non-wood forest commodities significantly differ from those of the low preference group. Thus, the general research objective aims to explore and investigate the factors involved in the public’s high preferences for non-wood forest products to promote the services in the country, based on the analysis of a national survey in the Czech Republic. The investigated factors are essential in fostering non-wood provisioning services as an alternative to timber production. The discourse on other potential contributions of transforming the activity from merely public recreation to becoming a viable income-generating activity is included, as the research findings are integral to assessing the promotion of non-wood provisioning services and aiming to enhance the multifunctionality of Czech forests (16–18), and support the development of the local bioeconomy.

## 2. Methodology

### 2.1. Study area

**Figure 2:**
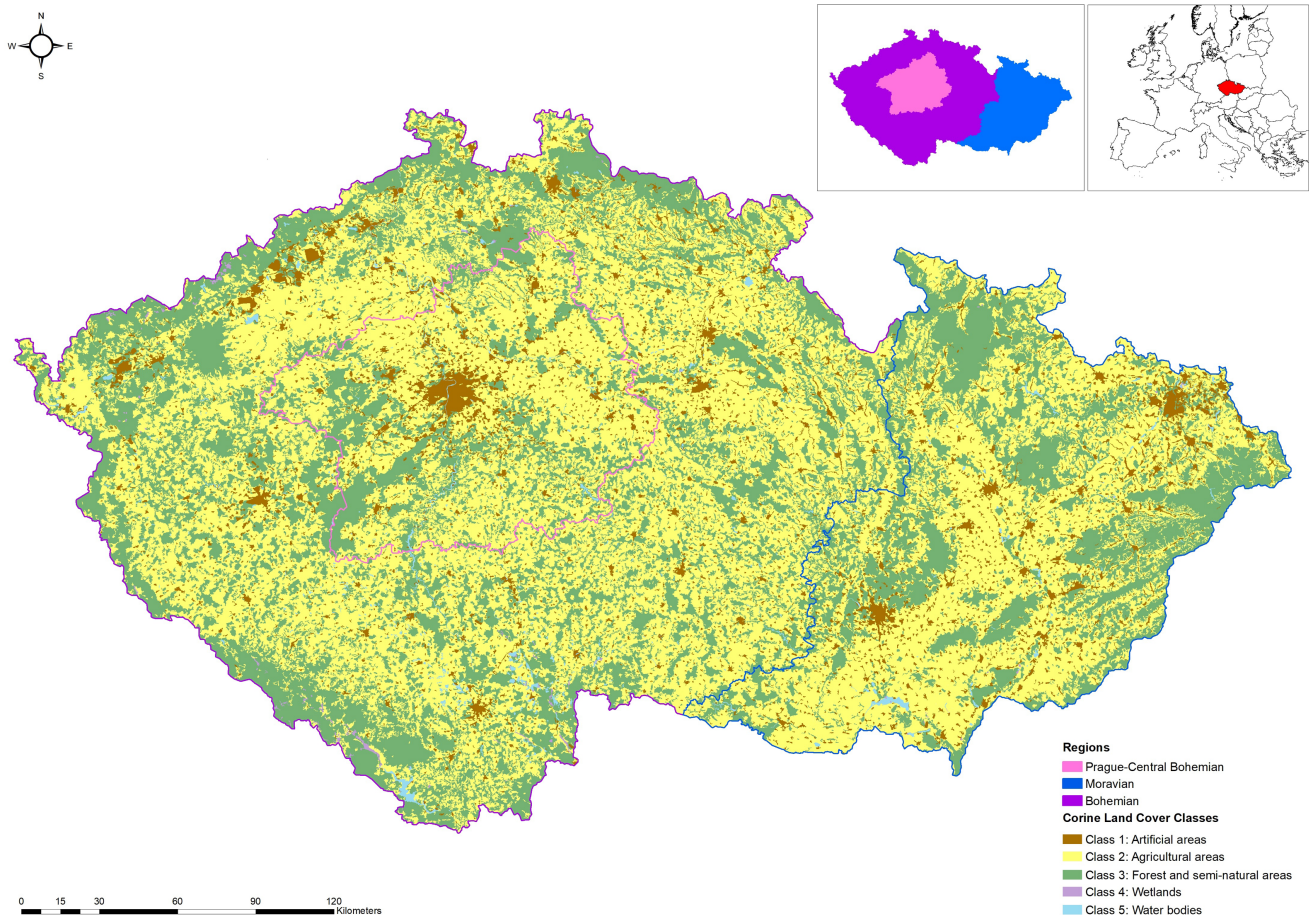
Map of the study area.

The Czech Republic is located in Central Europe, where its forested landscapes cover approximately one-third of the country’s total area (Figure 2). Approximately 54% of the forests are owned by the state. The entire Czech forest consists of 71.5% coniferous and 27.3% broadleaved trees, and 1.2% were without trees. Norway spruce [*Picea abies* (L.) Karst] is the predominant coniferous tree (50.0%), followed by pine (*Pinus* spp.) (16.2%), European larch (*Larix decidua* Mill.) (3.8%), fir (*Abies* spp.) (1.2%), and other coniferous (0.3%). Beech (*Fagus* spp.) is the primary broadleaved species (8.6%), followed by oak (*Quercus* spp.) (7.3%). Birch (*Betula* spp.) (2.8%) and other deciduous trees (8.7%) are also found in the Czech forests (33).

The country is divided into three study sites, i.e., Prague, the capital city and the region surrounding it (Central Bohemia), Bohemia, and Moravia. Originally, the Bohemian study areas were comprised of 10 regions [Nomenclature of Territorial Units for Statistics (NUTS) 3 level or *Kraj*] that also belonged to historical Bohemia, however, in this paper, the center part of the area (Prague and Central Bohemia) is considered as one separate study area because of their urban life and economic situation. Meanwhile, the Moravian study area combined three Moravian regions, similar to ancient times, and Moravia-Silesia (34, 39). In 2019, the Gross Domestic Product (GDP *per capita*) of the Bohemian study site ranged between approximately 13,000 to 18,500 euros, while Moravian GDPs were varying from about 16,000 to 20,000 euros. Furthermore, compared to the other regions, Prague and Central Bohemia had the highest GDP of about 20,000 and 46,500 euros (40). In this paper, the country is also presented by the division based on the results of a cluster analysis (high and low usage preferences) to effectively segment the areas and better target the promotional program.

### 2.2. Conceptual framework on preferences of non-wood forest products

Any product extracted from and provided by the forest is defined as forest provisioning service, including wood and non-wood forest products (2). Furthermore, the Food and Agriculture Organization’s (FAO) definition of non-wood forest products excludes all woody raw materials (41), and is used for the terminology in this paper.

**Figure 3:**
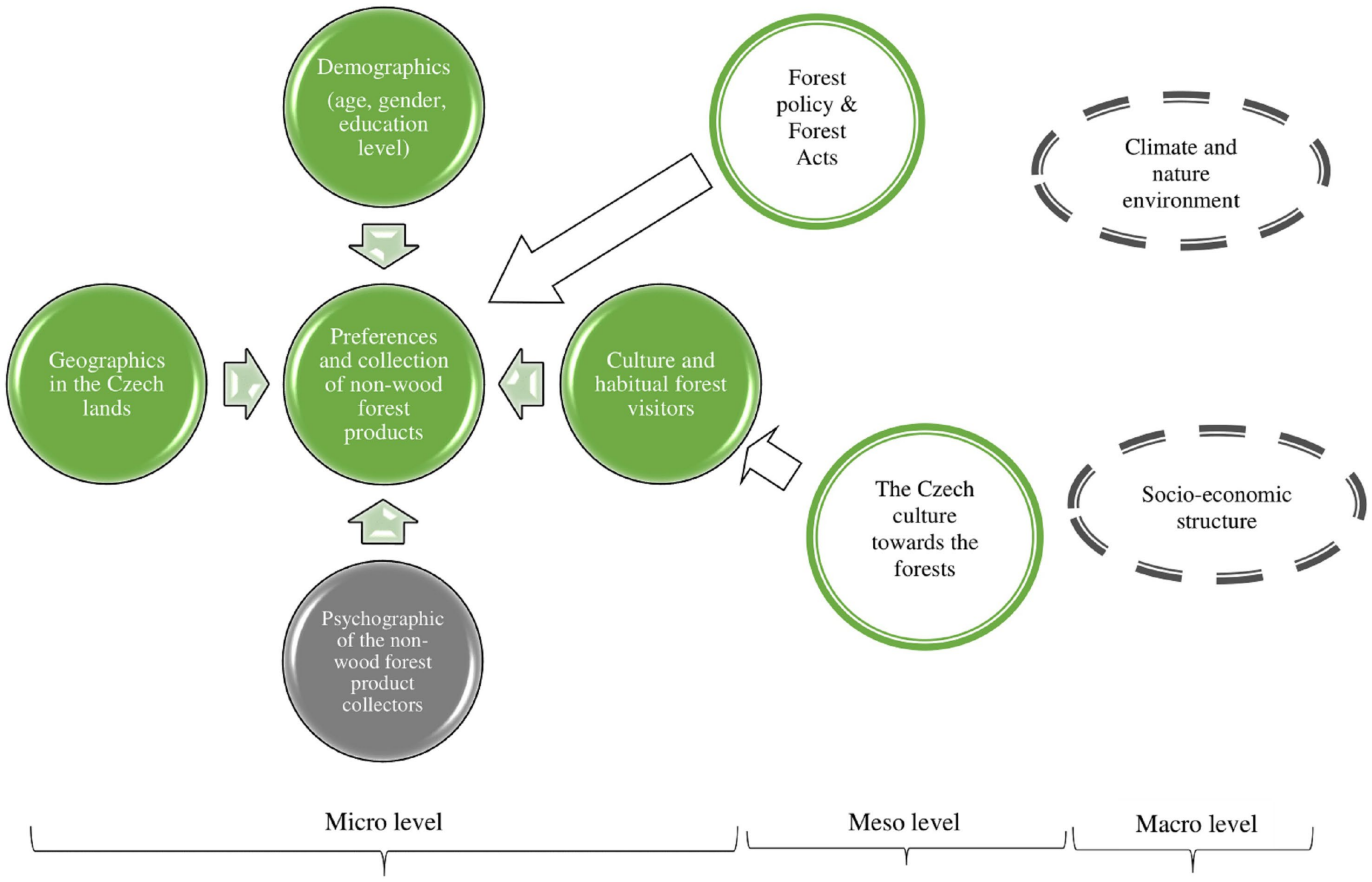
Conceptual framework of the non-wood forest product promotion, from recreational to potential income-generating activities.

Based on the non-wood forest products’ value (goods and services) networks that were categorized at the micro, meso, and macro levels (42), we developed a modified conceptual framework of the study (Figure 3). The framework aimed to answer the research question: what are the factors that influence society’s high preferences for non-wood forest products to promote the ecosystem service, from primarily focused on public recreation to an income-generating activity? The general research objective is to explore and investigate the factors involved in society’s high preferences for non-wood forest products to promote the services in the Czech Republic. Our hypotheses, the factors leading to society’s high favor for non-wood forest commodities are significantly differ from those of the low preference group. Therefore, the examined factors are essential in promoting non-wood provisioning services as a viable substitute for timber production.

The microscopy analysis of this paper was from the forager’s point of view, the societal aspect (Figure 3). The correlation pathway detailed in the framework was modified from the four basics of market drivers (43), which could affect preferences for non-wood forest commodities. The demographics of the respondents, such as age, education level, or gender, influenced the preferred utilization and type of favored commodities (19, 44). The cultural customs and habits of the Czech people encouraged them to visit forests for various recreational purposes, including gathering non-timber goods (34, 45). Furthermore, varying degrees of destruction caused by the bark beetle infestation in Czech forests (36, 38) can potentially decrease the frequency of forest visits and the collection of commodities in different regions (46) (Table 1). For this study, the psychographic factors were not included because we wanted to focus on the tangible aspects at the community level. The conceptual framework considered the potential direct link to the other factor level (42), although the meso and macro levels were not assessed in this paper. Nevertheless, the discussion acknowledges the influence of forest laws implemented in the country and the public’s view on the forests, which operates at the meso level (9, 19). The discussion also encompassed the contributions of non-wood forest commodities to the environment, economy, and other societal benefits, which are part of the macro level.

**Table 1:** Matrix of the preferences and collection of non-wood forest products (42, 43, 47) investigated in the Czech Republic.

Variables	Indicators	References
Preferences of non-wood forest products: high vs. low preference	Demographic condition Age Gender Education level	(19, 44)
	Culture and habit: frequency of forest visit	(34, 45)
	Geographic condition of the Czech lands: various level of bark beetle’s destructions	(36, 46)
Preferences of non-wood forest products: mushrooms	Amount of mushroom collection	(35, 48)

The national non-wood forest products information was comprised of forest mushrooms (reported as the total collection and not by species) and berries, i.e., bilberries, raspberries, blackberries, cowberries, and elderberries. Mushroom is the most gathered non-wood forest product in the Czech Republic (35, 48). Hence, for data comparison and validation, the volume of collected mushroom raw data gathered by the MoA during the summer of 2018 was used to reflect the last year’s activity of the 2019 respondents.

### 2.3. Survey data collection on the preferences of non-wood forest products

This study relied on the 2019 national survey data of public preferences for collecting the forest berries, mushrooms, honey, and medicinal herbs that were also used as the indicator of the forest non-wood provisioning services in the Czech Republic (Supplementary Figure SA.1). The respective questions in the questionnaire were developed as part of the project “Advanced research supporting the forestry and wood-processing sector’s adaptation to global change and the 4th industrial revolution (EVA 4.0)” carried out by the Faculty of Forestry and Wood Sciences, Czech University of Life Sciences, Prague, the Czech Republic. The questionnaire underwent an initial internal pretesting process before its finalization. A closed-ended questionnaire consisting of socio-demographic characteristics and preferences on non-wood forest products was obtained. Five-degree levels for the preferences were applied to the questionnaire, ranging from 1 (one) as most preferred to 5 (five) as least preferred.

The survey was done in collaboration with an external market research company, REMMARK, a.s. (Prague, Czech Republic), and results at the national level have been reported (24). A computer-assisted web interviewing (CAWI) technique was employed to recruit representative samples of online respondents. The samples were drawn in proportion to the population size per region (NUTS 3 level) and county, as specified by the national statistics office (49). The sample selection also ensured an equal representation of both genders among the respondents. The mandatory entry criteria for the respondents were a minimum age of 18 years old, allowing self-representation. The respondents were also required to have access to an internet connection, because the algorithm technique generated messages and sent the questionnaires to the potential participants through various online platforms. The respondents themselves would fill out the questionnaire. No private information was stored, thereby assuring the anonymity of the participants on the survey.

The survey was completed upon achieving the minimum required sample size for a national representation (1,000 responses), which was reappraised by the following formula for population survey (50, 51):


$$n=\frac{Z^{2}*p*q*N}{\left(N-1\right)*e^{2}+Z^{2}*p*q}$$

where:

*n* = required sample size.

*Z* = z-value corresponding to the desired level of confidence (95%) = 1.96.

*p* = estimated proportion of the interested trait (preference of non-wood forest product) = 0.5.

*q* = 1 - p.

*N* = total population size = 10,670,000.

*e* = desired margin of error = 0.05.

Taking into account a design effect of two (due to the heterogeneity in regional population distribution), the minimum required sample size amounts to 776.

### 2.4. Data analysis

Statistical data analysis was performed to answer the general objective (factors influencing the preferences of non-wood forest products). The general characteristics of the respondents were analyzed using descriptive statistical analysis. The education attainment of respondents was categorized as high (college or university), medium (high school graduate with the standardized graduation certificate, a so-called maturita),^1^ and low level (without the graduation certificate). The study areas were divided into Bohemia, Prague-Central Bohemia, and Moravia, especially because Moravia experienced a significantly more deteriorating situation due to bark beetle attacks and the changes in volumes of non-wood forest products than Bohemia (32, 36–38). Categorical group comparison was performed using the chi-square test or Fischer exact test. Additionally, a K-means cluster analysis was implemented to classify the utilization of non-wood forest products regardless of the areas. Classification of the public preferences and areas was performed to better understand the situation for developing the services and policy recommendations. An area that indicates a potential market for non-wood forest products can be highlighted for investment priority and further development. Results of the respondents’ classifications were associated with the volume of collected mushrooms from the annual national collection of non-wood forest products in 2018 using the Spearman’s correlation analysis for non-categorical and not normally distributed data.

A binary logistic regression with a conditional forward approach was used to identify the potential predictors of high preferences in non-wood forest products (forest berries, mushrooms, honey, and herbs). The answers “neither,” “rather not,” and “certainly not” were grouped as low preference and coded as 0 (zero), while high preference was defined as a combination of “certainly yes” and “rather yes” and coded as 1 (one). The potential independent covariates were based on the published research concerning the drivers and predictors of forest visits (19, 36, 44, 46), and the research interests for differentiating the study sites. Hence, the following independent predictors of having higher preferences for utilizing the non-wood forest product were included in the initial model: age, gender (1 = female), education level (1 = graduation with the maturita and above), area (1 = the observed part: Prague-Central Bohemia/Bohemia/Moravia), and frequency of forest visits (1 = frequently, defined by at least monthly visit to the forests). The predictors considered the previous reports’ information presenting the situation of Moravia land that experienced significantly more deterioration due to bark beetle attack, especially after 2018 (32, 36, 37, 38). A *value of p* of less than 0.05 was designated as the statistical significance in all analyzes. IBM SPSS version 26 was used for all statistical analyzes (IBM Corp., Armonk, NY, United States). The Sankey diagram was developed using RStudio version 2022 (RStudio, PBC).

## 3. Results

### 3.1. Preliminary analysis for re-classification of the respondents

The nationwide survey in the Czech Republic was carried out in June 2019 using the CAWI technique. All responded questionnaires (100%) amounting to a total of 1,050 representative samples were included in the analysis of non-wood forest product preferences. The respondents were recruited proportionally based on population size by NUTS 3 (49) and gender (49.2% of respondents were female, *n* = 517). In total, mushrooms were the most preferred non-wood forest product, whereas 58.5% of the respondents reported definitely being willing to collect them. Forest berries came second after mushrooms (47.9%), followed by herbs (22.6%), honey (19.6%), and flowers (11.0%).

A K-means cluster analysis was employed to classify the respondents. Two groups of respondents were developed based on their uses of the entire non-wood forest products and were asked in the questionnaire (forest mushrooms, berries, herbs, honey, and flowers), i.e., higher and lower utilization. All non-wood provisioning services depicted significant differences between the high and low preference classes (Appendix Tables SA.1, SA.2; Supplementary Figure SA.2). Supplementary Figure SA.3 shows the diagram flow in clustering the respondents based on their choices in utilizing non-wood forest products.

**Figure 4:**
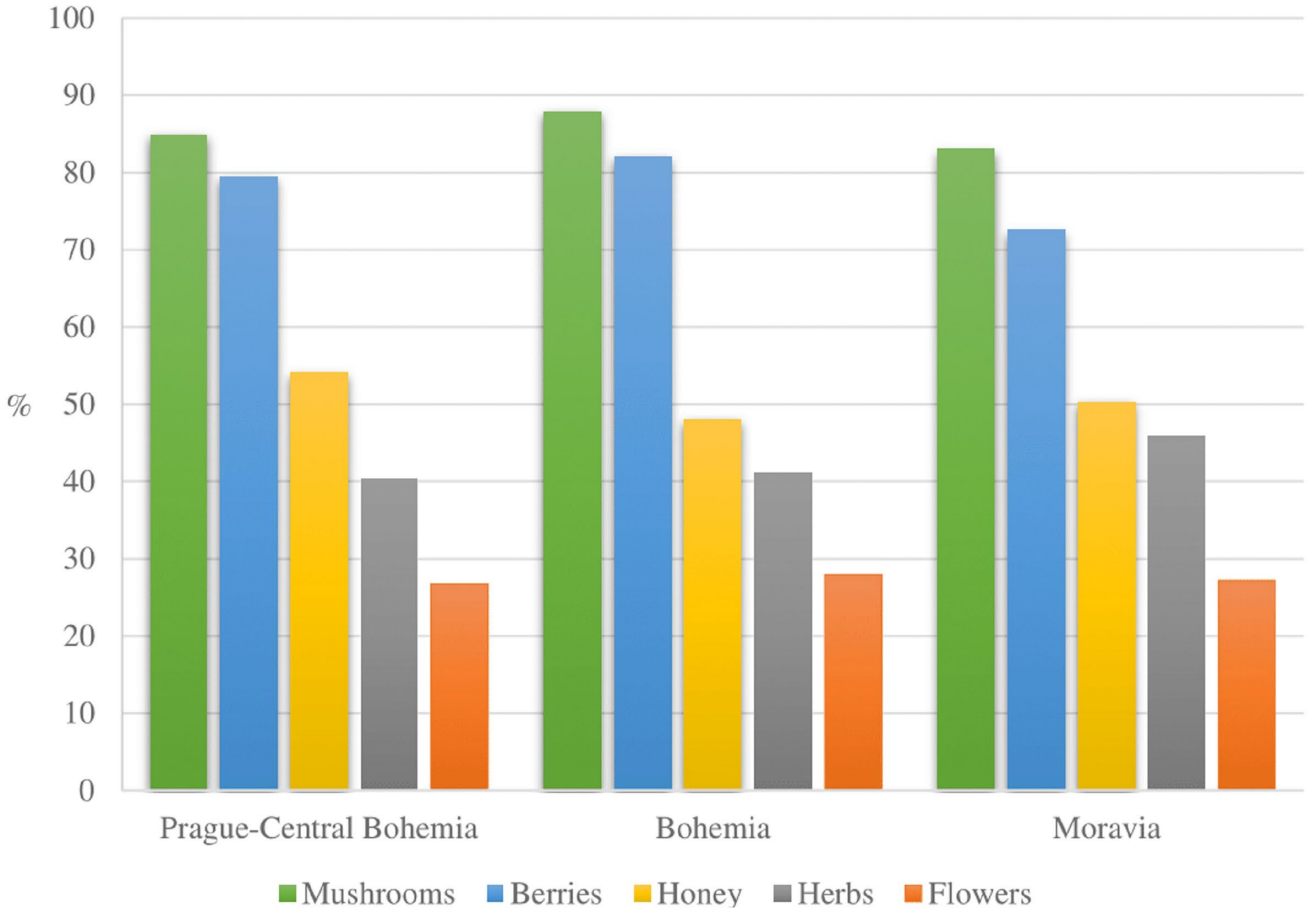
Proportion of respondents favoring the non-wood forest products by study sites.

Figure 4 presents the proportions of respondents who answered that they would utilize the non-wood forest products in between the study sites. There are no significant differences in the preferences for non-wood forest commodities between the study sites. Mushrooms were the most favored commodities, followed by berries in all study sites. The preference for herbs collection was the highest among the Moravian respondents, while honey was more utilized by the Prague-Central Bohemian participants.

### 3.2. Main factors contributing to the high preference of non-wood forest commodities

According to the new classification, 55.9% of the Bohemian respondents were categorized to have a higher preference for using non-wood forest products, followed by the Moravian (30.6%) and Prague-Central Bohemian participants (13.5%), but no statistically significant differences were found when compared to the lower preference group (*p* = 0.163; Table 2). Respondents who less preferred to use the non-wood forest products were significantly older (44.9 ± 13.4 years) than their counterparts (40.7 ± 13.2 years; *p* < 0.001). Generally, more frequent forest visitors were revealed to utilize more non-wood forest products than the less frequent ones (*p* < 0.001).

**Table 2:** General characteristics of the respondents (*N* = 1,050)^1^.

Characteristics	Preference of non-wood forest product utilization		Value of *p*
	Lower (*n* = 420)	Higher (*n* = 630)	
Study sites			
Prague-Central Bohemia	14.5 (61)	13.5 (85)	
Bohemia	50.0 (210)	55.9 (352)	0.163
Moravia	35.5 (149)	30.6 (193)	
Gender (female)	46.9 (197)	50.8 (320)	0.231
Age (years)	44.9 ± 13.4	40.7 ± 13.2	<0.001
Education level			0.438
High school without the secondary school leaving certificate and below	47.1 (198)	48.7 (307)	
High school with the standardized matriculation certificate (maturita)	35.5 (149)	36.8 (232)	
Higher education	17.4 (73)	14.4 (91)	
Size of the municipality of residence (inhabitants)			0.898
Less than 5,000	35.7 (150)	37.8 (238)	
5,000 – < 20,000	18.8 (79)	17.8 (112)	
20,000 – < 100,000	21.4 (90)	21.6 (136)	
≥ 100,000	24.0 (101)	22.9 (144)	
Frequency of forest visits			<0.001
Several times per week	8.8 (37)	22.4 (141)	
Once a week	20.2 (85)	31.4 (198)	
Once a month	35.5 (149)	31.7 (200)	
One or two times per year	33.6 (141)	14.0 (88)	
Less than once a year or never	1.9 (8)	0.5 (3)	

^1^Data are presented in mean ± sd or % (*n*); Fischer exact or chi-square test was used for analyzing categorical data, while the Independent t-test was used for comparing the age of the respondents; significant level: *p* < 0.05.

Bohemia has an area of approximately 4.6 million ha, with almost 1.6 million ha being forested landscape, which is more than double that of the Moravian forest area (about 0.7 million ha). Prague and Central Bohemia possess much fewer forest areas (± 0.3 million ha). The proportions of age group, gender, and education level of the respondents in the two areas were similar. Approximately 65% of the total respondents were from the productive age group (25–54 years). Nearly 60% of them had the standardized graduation certificate (maturita), and roughly 95% visited the forests at least once a year. All Prague-Central Bohemian participants lived in a highly populated area (more than 100,000 inhabitants), while the respondents from the other study sites resided in the lower population size (*p* < 0.001). Significantly more Prague-Central Bohemian dwellers also held a higher education level than the Bohemian and Moravian respondents (29.5, 11.6, and 16.4%, respectively, *p* < 0.001). All respondents in the three study sites often visited the forests on a weekly basis. Based on the national data of collected non-wood forest commodities in 2018, in the entire Bohemian region, the volume collection of forest mushrooms (17,490 tons) and bilberries (7,870 tons), of which almost 7,000 and 3,000 thousand tons of mushrooms and bilberries, respectively, were gathered in Prague-Central Bohemia. Meanwhile, the collection of mushrooms and bilberries in the Moravian site was 14,940 and 3,560 tons, respectively.

Table 3 presents the final model of predictor analysis of the preference in collecting the non-wood forest products based on the binary logistic regression forward step approach. Since the investigation aimed to examine the opportunities for promoting these products, therefore number 1 (one) was defined as high preference for utilizing the commodities. The logistic regression analysis revealed that frequent forest visitors were the primary predictor of high utilization of all non-wood forest products. They had between 1.437 to 4.579 odd ratios of using the non-wood provisioning services. Young-aged participants were also more likely to gather forest berries, honey, flowers, and herbs. Female respondents were 1.333 more likely to pick the forest flowers. Furthermore, based on the study area, the Moravian respondents were less likely to harvest berries (*p* = 0.003). Contrarily, those respondents had a 1.433 odd ratio of using the forest herbs (*p* = <0.001) compared to the participants in the other study sites.

**Table 3:** Predictors of the high preference for non-wood forest product utilization.

Final model of the predictor analysis	Beta	Standard Error	Odds ratio (Exp B)	95% C.I	R^2^	Sig.
Forest mushrooms (1 = high preference)						
Frequency of forest visits (1 = frequently)	0.865	0.189	2.375	1.641–3.437	0.034	<0.001
Forest berries (1 = high preference)						
Region (1 = Moravia)	−0.469	0.160	0.626	0.457–0.857		0.003
Age of respondents (in years)	−0.024	0.006	0.976	0.965–0.988	0.098	<0.001
Frequency of forest visits (1 = frequently)	1.073	0.166	2.924	2.112–4.049		<0.001
Forest honey (1 = high preference)						
Age of respondents (in years)	−0.020	0.005	1.020	1.011–1.030	0.031	<0.001
Frequency of forest visits (1 = frequently)	0.362	0.150	1.437	1.072–1.926		0.015
Forest flower (1 = high preference)						
Age of respondents (in years)	−0.025	0.005	1.026	1.015–1.036	0.079	<0.001
Frequency of forest visits (1 = frequently)	1.041	0.202	2.831	1.905–4.207		<0.001
Gender (1 = female)	0.288	0.143	1.333	1.008–1.764		0.044
Forest herbs (1 = high preference)						
Region (1 = Moravia)	0.360	0.164	1.433	1.039–1.976		0.028
Age of respondents (in years)	−0.014	0.006	0.986	0.975–0.997	0.135	0.014
Frequency of forest visits (1 = frequently)	1.521	0.187	4.579	3.173–6.608		<0.001

*N* = 1,050, *R*^2^ value was obtained using Nagelkerke *R*^2^.

No statistically significant difference was observed between the new cluster groups concerning the volume of forest mushroom collection. Additionally, there was a negative correlation between public preference for mushroom collection and the volume of *the gathered* mushrooms (*r*^2^ = 0.100, *p* = 0.018).

## 4. Discussions

Non-wood forest products are essential for achieving the SDGs including poverty reduction (SDG 1), alleviating hunger (SDG 2), improving health and wellbeing (SDG 3), promoting decent work opportunities (SDG 8), fostering responsible production and consumption (SDG 12), addressing climate change (SDG 13), and ensuring sustainable life on Earth (SDG 15) through their connection to forests. They also play a crucial role in advancing the European policy priorities of the multifunctionality of the forests (52).

The utilization of plant-based non-wood forest products varies across different countries and is influenced by their cultural practices and habits. Northern European countries, such as Finland, Sweden, and Norway, have a strong tradition of utilizing the commodities due to their abundant forests. Wild berries, such as lingonberries, cloudberries, and bilberries, are highly valued in this region (53–55). Berries are used in various food products such as jams, juices, desserts, and traditional dishes. In addition to berries, mushrooms are also popular in Northern Europe. The most favorite edible fungi species are chanterelles and boletes (56). Studies have shown that mushrooms, especially truffles, are highly preferred non-wood forest commodities in Southern European countries such as Italy, France, and Spain (57, 58). The culinary traditions associated with truffle consumption have led to high demand and economic importance (59–61). In addition to mushrooms, Mediterranean countries also utilize wild herbs, aromatic plants, and medicinal plants. These plants are commonly used in traditional cuisines and herbal remedies (31). In addition, corks, chestnuts, berries, and acorns are included in the popular non-wood forest products in Mediterranean countries (30). Central European countries include a mix of preferences for non-timber products due to their geographical location and cultural diversity. In Germany, for example, Christmas trees are considered an integral part of non-timber commodities (62). The Czech Republic has a strong tradition of mushroom picking (48). The Czech population also favors foraging forest berries (34, 35). Comparable with the Czech Republic, forest mushrooms and fruits (especially bilberries) are two of the most collected non-timber commodities in Poland (63). In Slovenia, a notable consumption of honey, indicates the country’s affinity for this natural product derived from its forest, thereby emphasizing honey as a noteworthy non-timber product (64).

### 4.1. Key factors predicting the high preference for non-wood forest products

According to Table 2, the majority of the respondents (77.2%) visited forests at least once a month, which was higher compared to the 2006 national survey (53%) (65). Among the Czech population, visiting forests for recreational purposes and engaging in various activities is a significant driver, with most individuals enjoying the outdoor environment or just walking (45). Visiting forest at least once a month was found to increase the preferences of the respondents (between 1.4 to 4.6 odd ratios) in utilizing all non-wood products, i.e., mushrooms, berries, honey, flowers, and herbs, combined with the younger-aged respondents for berries, honey, flowers, and herbs (Table 3), indicating the forests are visited by relatively physically active attendees. In contrast to the negative effect of the COVID-19 pandemic on the national economy in general, a considerable increase in the number of forests visits was observed, as confirmed by several studies in Europe, such as in Germany (66) and Slovakia (67). A study in the Western Italian Alps also reported that forest cultural service was gaining more attention than the provisioning function after the pandemic (68), implying the potential recreational service development for improving human wellbeing. In the Czech Republic, a study by Jarský et al. (69) reported that the average forest attendance in 2020 (amidst the COVID-19 movement restrictions) in the selected forest sites increased significantly compared to 2019. Visitors’ characteristics and motivation for forest visits have changed toward appreciating recreation services. The dynamic of forest attendance was in line with the periods of strict restrictions. The same study, however, indicated that the collection of non-wood forest commodities did not change substantially, although the amount of collected prominent berries increased. Nevertheless, a substantial decrease in the number of visitors is anticipated in some forest areas during COVID-19 restrictions, which can lead to different actions of the forest owners. For instance, a decrease in forest visitors could alter wild animal behavior (70), which might negatively affect the growth of forest plants. The pandemic is also expected to cause a shift in public preferences and perception of the forest’s performance.

More natural forests and less human intervention were preferred by the Czech forest visitors (19). Given the alarming state of the Czech forested regions caused by the infestation of bark beetles, which increased logging activities considerably and damaged the landscape beauty of the Czech forests (71), and to gain a deeper understanding of the market situation of non-wood forest commodities in each study site, the survey respondents in the three areas were included as one of the covariates of the non-wood forest commodity preferences. Table 3 showed that the Moravian respondents were less likely to harvest berries (*p* = 0.003) than participants in the other study areas. The counterpart study areas, Bohemia and Central Bohemia, possess larger total forest areas of all vegetation types and a lower reduction of forest areas in 2018 than Moravia. Additionally, residents of urban areas, such as Prague, frequently engage in “nature excursions,” a popular recreational activity during weekends in the nearby countryside (34). The Moravian participants were also 1.4 times more likely to use the forest herbs compared to their two counterparts (*p* = <0.001), ostensibly because the areas consisted primarily of arable land (Figure 2), giving more space for the growth of herbs in the forest fringes. This is evident in some parts of Moravia, especially in the White Carpathians, which is also considered a high biodiversity area rich in natural herbs (72, 73). As a result, the Moravian inhabitants tended to use more natural remedies, including forest herbs (74), than the Bohemians.

Based on the analysis, gender played a role in selecting forest flowers, as the male participants had a 1.3 odd ratio not collecting them. The results implied the importance of targeting the forest visitors that might improve the attractiveness of the managed areas and business portfolio for the forest owners. Furthermore, like many other European countries in the Danube region, forestry in the Czech Republic, i.e., wood production, is considered a male-dominated realm (75). Meanwhile, women are more identified as the potential users of various non-wood forest products, primarily plant-based commodities, as reported in Switzerland and the Czech Republic (24, 44); thus, the promotion of these services could potentially involve more female forest owners and employees.

Results of correlation analysis showed that the higher preference scores in utilizing mushrooms were not supported by the volume of the gathered mushrooms indicating that assistance is required for developing activities in need of support. By clustering the respondents based on the high and low preferences in utilizing non-wood forest commodities, the promotion of this forest provisioning service can be better targeted, e.g., to develop the infrastructure in the location with a high choice in each study site. From the forest management point of view, the information about provisioning services brings new initiatives for innovation or diversification of the product and services portfolio, especially concerning recreational services. The development of business activities will lead to a strengthening of competitiveness and enhancement of the economic value of the forest. Moreover, innovative product portfolios may be less sought-after locations to make more attractive, thereby relieving over-touristed places. In this case, in the areas with a high demand for non-wood forest product picking activity, forest vegetation can be managed more sustainably to ensure and maintain the harvesting volumes as attractive attributes of the forests and, at the same time, ecological stability of the trees.

### 4.2. Potential contributions of non-wood forest product to the economy, environment, and society: theoretical implications

To date, the Czech Forest Policy (76) is the newest policy document in forestry that was introduced in 2020 to achieve “Forest for Society” by setting strategic actions in forest management from the perspective of economic, environmental, and societal values on a long term basis. Forest laws play a critical role in facilitating the transition of recreational activities of collecting non-timber goods to profitable income streams within the nation. Under the Czech Forest Act, free access to the forests is a public right, which is also included in national forest laws of other Central European countries, such as Germany, Poland, Slovakia, and Slovenia (9); however, the right to free access to forests is not universally guaranteed across Europe. As a result, the public’s relationship with non-wood forest products may be influenced by these rules. In the Czech Republic, most non-timber commodities such as forest mushrooms, berries, and herbs can be freely collected, and the majority of them are also gathered for personal consumption, often as part of recreational and relaxation activities (65). The collection of non-wood forest products is permitted in approximately 92% of the Czech forest areas (regardless of the ownership of the forests, except for military forests, heavily protected natural preserves and zones of national parks), with the caveat not to damage the forest environment (37, 9), which limit the income generating efforts from other than timber sales. In contrast, the collection activities in most North European and Mediterranean countries can be done only by licensed pickers approved by the local government and is free up to a specific quantity limit (77). However, Czech forest owners can still capitalize on extracting non-wood forest commodities by independently collecting and selling the products, thereby deriving benefits from their endeavors, especially if a market for the products can be established by private entities or local government, like in Finland (78) or Sweden (79).

Previous research reported that the collection of non-wood forest commodities is pursued by the public with different demographic and socio-economic backgrounds and various motivations. Yet, they do not aim to ensure household sustenance, but mainly for domestic consumption, concentrating less on economic purposes. Therefore, currently, they are not managed professionally, and the collection activities depend on each individual’s preferences and motivations (34, 35, 48). Of all non-wood forest commodities, mushrooms were the most preferred products in the Czech Republic, followed by forest berries (Figure 4). The findings agreed with the national forestry report, where mushrooms were also reported as the most foraged non-wood forest products in 2018 and 2019, by 16.5 and 21.9 thousand tons, respectively. The total collected forest berries in the same observed years were 11.4 and 9.8 thousand tons, respectively. The market price of forest mushrooms in 2018 and 2019 was about 6.9 and 6.3 euros per kg, respectively. Meanwhile, the forest berries varied between 5.7 to 8.1 euros per kg. The potential economic values of forest mushrooms and berries in the observed year were estimated at 113–138 million and 64–67 million euros, respectively, indicating the possible economic benefits of the commodities for the forest owners (33). No information was available about the other products, e.g., forest honey, flowers, and herbs, implying that their potential is still undervalued. A study by Lovrić et al. (80) estimated that non-wood forest commodities create a total economic value of 23.3 billion euros per year in Europe. The Czech Republic is among the countries with the highest collection share. In addition to the plant-based collection, hunting is also a recreational activity in the country, even though it is less practiced by the general public and favored mostly by male forest visitors (24, 45).

In response to the current reduction of the forested vegetation regions due to bark beetle infestation, especially in the coniferous forests, shrub planting is recommended as an alternative solution for improving the environment by increasing greener areas because berries have a short growing period compared to the wooden trees (81). Mixed-tree silviculture can also be promoted more vigorously in the country, such as provisioning seed supplies or government financial support in providing incentives for the owners that manage such forests for environmental benefits. Bilberries can be found in forests with many Norway spruce and silver-fir (*Abies alba* Mill.) trees (63, 82) or oak woods and upland heath (83). Blackberries grow under the silver-fir-dominated vegetation type (84), while raspberries are associated with fir-beech mixtures (85). Furthermore, cowberries grow in coniferous forests, especially with Scots pine (*Pinus sylvestris* L.), light deciduous oak forests, and on pristine and drained peatland (86). Unlike other forest berries, elderberries can grow in moderate to highly eutrophic and disturbed soils and are less commonly found within forests because they need high light intensity (87). Berries are categorized as shrubs (88). Soil with shrubs contained higher nutrients than open and non-shrub soil areas, demonstrated by higher organic carbon and nitrogen content in the 0–10 cm soil profile (89). Another interaction of soil and berries, especially those belonging to the genus *Vaccinium,* showed an indication of potential microbial community structure occurring in root-associated bacteria of *Vaccinium angustifolium* (wild blueberry), which play an important role in soil fertility resulting in beneficial plant–soil interaction (90). Meanwhile, various edible mushrooms, e.g., porcini (“hřib smrkový” *Boletus edulis*) and morels (“smrž obecný” *Morchella esculenta*) can grow in different types of forest, e.g., mixed forests dominated by Norway spruce and oak. Some edible mushrooms from the Boletus family can also grow on grassy land with high light exposure (87, 91). For forest mushrooms, richness is highly dependent on the diversity of the stands and the association with deadwood (91–93). The uncut-drying trees, caused by insect infection, can be used as the habitat of some edible mushrooms that are potentially economically valuable for forest owners. The presence of deadwood in the forests can also attract forest visitors and is considered to be more natural (19). Additionally, the measures taken to mitigate and adapt to climate change also suggest that alterations in the composition of tree species could lead to distinct ecological conditions. This, in turn, has the potential to enhance or jeopardize the production of certain non-wood forest commodities, posing a challenge to overall development.

As alternative commodities to wood, the Czech forest visitors proficiently collect and utilize non-wood forest products for personal use. One of the primary values of these plant commodities is perceived to be more natural than commercial ones in a managed field; hence, they can be more beneficial for human consumption and health. However, the usage of plentiful forest herbs in the country is still underestimated (74). Recently, the health benefits of forest plants have become increasingly acknowledged. It is evident that several essential minerals of forest bilberries (*V. myrtillus* L.), such as, macroelement potassium (K), phosphorus (P), and calcium (Ca) as well as microelement iron (Fe), were discovered to be superior [approximately 572 mg K, 117 mg P, 140 mg Ca, and 1.8 mg Fe per 100 g dry matter (DM), respectively] than their counterparts that grew in open sites (about 508 mg K, 110 mg P, 120 mg Ca, and 1.7 mg Fe per 100 g DM, respectively) or commercial plantations (approximately 548 mg K, 64 mg P, 24 mg Ca, and 0.8 mg Fe per 100 g DM, respectively) in Italy (94). Hence, 100 g forest bilberries cover between 13 to 34.3% of daily recommended intake of selected minerals for the Czech healthy adults (95). Various wild berries from European forests demonstrated notable concentrations of vitamin C. For instance, vitamin C analysis on wild raspberries (*R. idaeus* L.) reported that they contained about 45 mg (96), surpassing the range found in diverse raspberries (7.4 mg to 36.0 mg/100 g fresh weight) (97). Meanwhile, vitamin C of wild blackberries (*R. fruticosus* L.) consisting of about 13 mg and 33 mg (96, 98) were comparable to the range in a variety of blackberries (10.3 mg to 70.4 mg/100 g fresh weight) (97). The published research findings support their nutrient-dense attributes.

*B. edulis* wild mushrooms could present high concentrations of K, P, sodium (Na), and magnesium (Mg) with mean concentrations of approximately 790 mg, 240 mg, 520 mg, and 80 mg per 100 g DM, respectively, even when grown in thin soil in Polish forests (99); all of these values surpassed those of cultivated mushrooms *Agaricus bisporus* (100). Various wild mushrooms from European forests also exhibit high levels of iron or zinc (Zn); for instance, *Lactarius deliciosus* comprised between 8.1 to 12.9 mg Fe per 100 g DM, while ±17.0 mg of Zn per 100 g DM was found in *B. edulis* (101, 102). Consumption of 100 g wild *B. edulis* or *L. deliciosus* covers at least 50% of the recommended Fe and Zn per day for well-nourished adults (95). Some forest mushrooms (e.g., *B. edulis*, *L. deliciosus*) grown in the Czech Republic have higher protein per 100 g edible weight (mean: ±5.6 g) than common green leafy vegetables (103) or the cultivated mushrooms *A. bisporus* (2.9 g) (100). The protein content of 100 g forest mushrooms covers about 10% of recommended daily intake for well-nourished adults having a mean body weight of 70 kg (104, 105), indicating the their potential as a viable source of plant-based protein product. The nutritional profile of forest mushrooms and berries suggested that non-wood forest products have applications beyond their typical use for food consumption and recreation activity. Another recognized example of the benefits from these commodities is the biomedicine of various products made from non-wood forest materials, such as cough syrup made from spruce (*P. abies* L.) or ivy (*Hedera helix* L.), which are more efficient in cough treatment than allopathic medicinal products (106).

### 4.3. Research limitations and future implications

This paper focused on the preferences of the non-wood forest product collectors and did not specifically include and analyze the opinions of the forest owners and managers, which is considered one of the research limitations. Our paper presents data from 2019 before COVID-19 and thus can be understood as a “normal” year. The results were, on the other hand, more affected by the ongoing bark beetle calamity and its consequences. Further investigation is suggested from the forest owners/managers, especially after the bark beetle outbreak and the COVID-19 pandemic, because changes in viewing forest cultural services are expected. In the Czech Republic, average forest attendance during the COVID-19 restrictions in the selected forest sites increased significantly compared to 2019 (69). Drivers for forest visits have changed toward more appreciating recreation services, including collecting non-timber goods, yet the volume of gathered primary berries did not change substantially. Understandably, particular forests may experience a significant decrease in visitor numbers during restrictions, which could prompt different actions from forest owners. While studies in other European countries like Italy, Germany, and Greece have examined the impact of the pandemic on ecosystem services, these studies may not be directly relevant to the specific situation in the Czech Republic. Furthermore, these studies primarily focused on “urban green areas” rather than forests, as exemplified by the study conducted in Italy (107) and Greece (108). Concurrently, the study conducted in Germany focused on urban and peri-urban forests, and their findings confirmed a rise in the frequency of forest visits as well as the significance of forests in promoting well-being (109). Hence, research evaluating the shift in public preferences regarding forest ecosystem services following the pandemic is recommended to support our present findings further.

Since the research is carried out using the CAWI method, then only respondents who possessed equipment with an internet connection could participate. According to the Statistical Office, 85% of Czech population aged 16 to 64 years had internet access during the survey period (110), implying a high proportion of the potential target population above 18 years in our survey. Moreover, our study covered a larger geographic region (the number and sample structure of the respondents is representative, and the results can be projected for the whole country). The survey findings were also worth consideration for countries with similar natural conditions and experiencing similar disturbances (e.g., bark beetle). Online surveys have become increasingly utilized in the country, especially after the COVID-19 pandemic, which decreases the difficulties in getting permission for face-to-face interviews and consumes less time and resources.

Our results also revealed that similar amounts of mushrooms were gathered in areas with both high and low preferences for utilizing non-wood forest products. This finding initially aimed to identify specific areas with untapped potential. However, it is essential to acknowledge that the increased number of visitors to these forest sites may lead to overexposure. Therefore, it is recommended to consider implementing payment for ecosystem services (111) to mitigate the overuse of forest sites. Furthermore, by harnessing the potential of plant-based non-timber products for innovative applications like pharmaceuticals, cosmetics, and more (106, 112–114), including the utilization of various plant components apart from the fruits, can contribute significantly to advancing the forest bioeconomy.

Our research highlights a need for monitoring the shift in societal perception of forest visitors to support future forest management practices. These strategies may involve forest owners engaging in self-collection and sales of valuable non-timber goods, as well as promoting greater participation of female workers in the production of these commodities. To support development, identifying opportunities and challenges for establishing or enhancing local market infrastructure is encouraged. In our paper, the discussion about potential contributions of non-wood forest products to the economy, environment, and its societal benefits is based on a theoretical analysis derived from a scientific literature review. Therefore, it is important to note that certain deviations may occur once the promotion is implemented. For example, climate change leads to the necessity of modifying the tree species composition, which means that certain forest sites may no longer be suitable for the production of specific non-wood forest goods as they were previously. Conversely, newly structured forest areas may present higher potential for other commodities. Additionally, anticipated changes in national legislation aligned with the European forest policy are also expected to incentivize forest owners to prioritize the provision of non-timber commodities.

Based on the current findings, we also proposed comprehensive cost–benefit research, likely in a case study area. The recommended model should include not only the economic benefits of the non-wood forest products but also ecological and societal considerations, such as policy restriction and optimum collection, to provide a clear understanding of the implications of the potential activities. Promotion of the activities, as part of the provisional or cultural services of the forests, is expected to support the local bioeconomy.

## 5. Conclusion

This paper aims to present findings on the non-wood forest product preferences in the country as part of a baseline assessment for promoting the usage beyond a recreational interest, i.e., potential livelihood opportunities, and in addition, attracting forest visitors toward more environmentally friendly behavior. Two clusters of non-wood forest products were developed based on the preferences of the respondents. With various benefits from the non-wood forest products for society, the environment, and as potential income-generating activities besides timber production, the findings of the research project, dealing with socio-demographics of forest visitors, type of owners, and location of the property, can assist policymakers in the Czech Republic in making an appropriate resource allocation decision to promote the services strategically. For instance, mixed forests can be further supported by improving seed availability in the market, especially in heavily damaged areas. In the sites with a high demand for non-wood forest products, various berries, plants/herbs can also be provided as an income-earning alternative. Promotion of the activities, as part of the provisional or cultural services of the forests, is expected to support the local bioeconomy; however, it should still follow the national forest policy and laws that ensure the public’s right to access the forests.

## Data availability statement

The datasets presented in this article are not readily available because the mushroom aggregate data are published annually in the national forestry report (Green report) and publicly available from the Ministry of Agriculture of the Czech Republic website, https://eagri.cz/public/web/mze/lesy/lesnictvi/zprava-o-stavu-lesa-a-lesniho/. However, the national survey data are not publicly available. Requests to access the datasets should be directed to MHá (hajek@fld.czu.cz), RP (purwestri@fld.czu.cz).

## Ethics statement

Ethical approval was not provided for this study on human participants because this study relied on the 2019 national survey data of public preferences (*N* = 1,050) for collecting the forest berries, mushrooms, honey, and medicinal herbs that were also used as the indicator of the forest non-wood provisioning services in the Czech Republic. The survey was carried out in collaboration with an external market research company, REMMARK, a.s. (Prague, Czech Republic), and results at the national level have been reported (24). A computer-assisted web interviewing (CAWI) technique was employed to recruit representative samples of online respondents between 18 to 65 years old based on age, gender, education level, region, and municipality size, proportionally. The technique generates emails and sends the questionnaires to the potential participants through various platforms available online. The respondent him/herself would fill in the questionnaire. No private information was stored, and the anonymity of respondents in this survey was applied. The patients/participants provided their written informed consent to participate in this study.

## Author contributions

RP: conceptualization, methodology, software, investigation, formal analysis, data curation, writing–original draft preparation, and writing–review. MHo: conceptualization, investigation, writing–original draft preparation, and writing–review. MHá: conceptualization, methodology, writing–original draft preparation, writing–review, and supervision. PP and VJ: investigation, data curation, writing–original draft preparation, and writing–review. DH-B: software, investigation, formal analysis, data curation, writing–original draft preparation, and writing–review. MP and SG-J: investigation, writing–original draft preparation, and writing–review. BL: formal analysis, writing–original draft preparation, and writing–review. MR: methodology, investigation, data curation, writing–original draft preparation, writing–review, and supervision. All authors contributed to the article and approved the submitted version.

## Funding

This research work was supported by the Operational Program Research, Development, and Education, the Ministry of Education, Youth and Sports of the Czech Republic, with the grant no. CZ.02.1.01/0.0/0.0/16_019/0000803 (EVA 4.0).

## Conflict of interest

The authors declare that the research was conducted in the absence of any commercial or financial relationships that could be construed as a potential conflict of interest.

## Publisher’s note

All claims expressed in this article are solely those of the authors and do not necessarily represent those of their affiliated organizations, or those of the publisher, the editors and the reviewers. Any product that may be evaluated in this article, or claim that may be made by its manufacturer, is not guaranteed or endorsed by the publisher.

## Footnotes

^1^Maturita is a standardized graduation exam for upper secondary education, a pre-requisite for college and university studies in the Czech Republic.
