# Supplementary material for: From recreational to income-generating opportunities: assessment of public preferences for non-wood forest products in the Czech Republic
Source: Front Nutr. 2023 Sep 18;10:1193203. doi: 10.3389/fnut.2023.1193203 (PMC10545864; doi:10.3389/fnut.2023.1193203)
Supplement: Supplementary file 1 [file Data_Sheet_1.docx]

**Appendix:**

**Table A.1. Descriptive information of the preferences in using the non-wood forest products (n=1050)**

| Non-wood forest products | Mean | SD | Minimum | Maximum |
| --- | --- | --- | --- | --- |
| Mushrooms | 4.34 | 0.95 | 1.00 | 5.00 |
| Berries | 4.15 | 1.05 | 1.00 | 5.00 |
| Honey | 3.25 | 1.38 | 1.00 | 5.00 |
| Flowers | 2.58 | 1.33 | 1.00 | 5.00 |
| Herbs | 3.20 | 1.25 | 1.00 | 5.00 |

**Table A.2. Initial and final cluster centers**

| Clusters | Initial | cluster | Final | Cluster | *p*-value |
| --- | --- | --- | --- | --- | --- |
| Preferences | 1 | 2 | 1 | 2 |  |
| Use mushrooms | -3.54 | 0.68 | -0.56 | 0.37 | <.001 |
| Use berries | -3.01 | 0.82 | -0.77 | 0.51 | <.001 |
| Use herbs | -1.76 | 1.44 | -0.76 | 0.51 | <.001 |
| Use honey | -1.64 | 1.27 | -0.71 | 0.47 | <.001 |
| Use flowers | -1.19 | 1.82 | -0.67 | 0.45 | <.001 |

*Data comparison*

**Figure A.1. Flowchart of data collection and analysis**


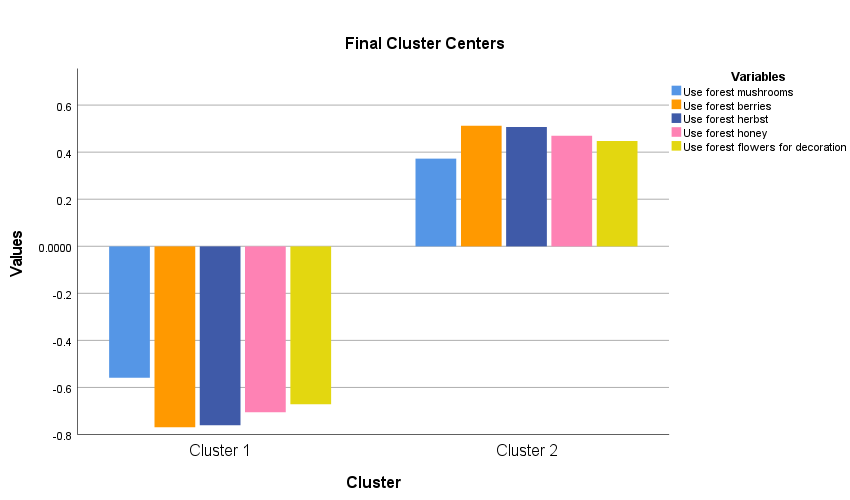


**Figure A.2. Results of K-means cluster analysis**


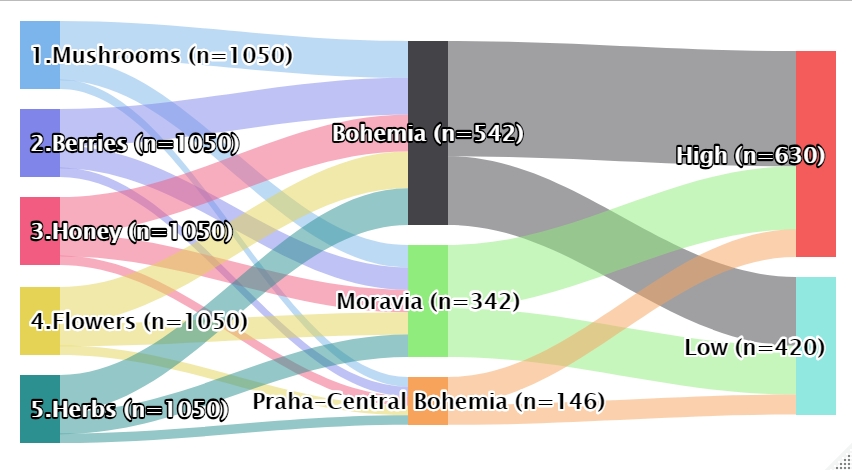


**Figure A.3. Diagram flow of the public preferences (high and low) cluster analysis in utilizing non-wood forest products in the Czech Republic (N=1050)**
